# Web-Based Learning for Children in Pediatric Care: Qualitative Study Assessing Educational Challenges

**DOI:** 10.2196/10203

**Published:** 2018-09-25

**Authors:** Gunilla Lööf, Nina Andersson-Papadogiannakis, Klas Karlgren, Charlotte Silén

**Affiliations:** 1 Department of Learning, Informatics, Management and Ethics Karolinska Institutet Stockholm Sweden; 2 Department of Paediatric Anaesthesia and Intensive Care Karolinska University Hospital Stockholm Sweden; 3 Department of Women’s and Children’s Health Karolinska Institutet Stockholm Sweden

**Keywords:** child, parents, learning, education, internet, information, preparation, preoperative, anesthesia, risk, delivery of health care, mobile phone

## Abstract

**Background:**

Hospitalization is a significant and stressful experience for children, which may have both short-term and long-term negative consequences. Anaesthesia-Web is a Web-based preparation program that has been well received and is being used worldwide to reduce stressful experiences, increase understanding, and exchange information in pediatric care. A deeper theoretical and educational understanding encompassing children’s learning processes on Anaesthesia-Web may optimize and support the development and design of similar websites for children in pediatric care.

**Objective:**

The objective of this study was to elucidate key educational principles in the development and design of websites for children in pediatric care.

**Methods:**

A directed qualitative content analysis was applied to analyze the content and design of Anaesthesia-Web from a theoretical and educational perspective. preunderstanding, motivation, learning processes, and learning outcome were used to analyze the learning possibilities of Anaesthesia-Web for children prior to contact with pediatric care.

**Results:**

We found 4 themes characterizing children’s learning opportunities on Anaesthesia-Web in the analysis: “In charge of my learning”; “Discover and play”; “Recognize and identify“; and “Getting feedback”. The analysis showed that Anaesthesia-Web offers children control and enables the use of the website based on interest and ability. This is important in terms of motivation and each child’s individual preunderstanding. Through discovery and play, children can receive, process, and apply the information on Anaesthesia-Web cognitively, emotionally, and by active participation. Play stimulates motivation and is very important in a child’s learning process. When facing pediatric care, children need to develop trust and feel safe so that they can focus on learning. On Anaesthesia-Web, children can recognize situations and feelings and can find someone with whom to identify. Several features on the website promote feedback, which is necessary to judge learning achievements, confirm understanding, and embody the need for repetition.

**Conclusions:**

Web-based preparation programs are important learning resources in pediatric care. Content and design needs to change from simply providing information to embracing the importance of a child’s need to process information to learn and fully understand. By developing Web-based preparation programs that include educational principles, Web-based technology can be used to its fullest advantage as a learning resource for children. The 4 educational themes described in this study should help future similar website developments within pediatric care.

## Introduction

### Web-Based Technology to Prepare Children for Anesthesia and Surgery

The internet is a rapidly emerging source of health service and health care information [1]. Web-based technology has been shown to efficiently convey information in a number of health areas [2-7]. Hospitalization is an important area where children require preparation because the event constitutes a significant and stressful experience, which may cause psychological and behavioral consequences and complicate cooperation and treatment as well as future dealings with medical services [8-11]. Anesthesia and surgery are some of the most stressful events for children while in hospital [12,13]. In terms of impact, children with preoperative anxiety and stress are at higher risk of developing postoperative excitement, distress, nausea, increased levels of pain and analgesic exposure, and delayed hospital discharge during the early postoperative period. Many children also show late reactions in the form of nightmares, separation anxiety, eating disorders, and temper tantrums within the weeks following anesthesia and surgery [9,10,12,14,15]. Preparation for a forthcoming hospitalization is important to decrease children’s distress and anxiety for medical procedures [13,16-18]. Adequate preparation is also important to generate accurate expectations and to reduce uncertainties and inconsistencies between fantasy and reality [11,16-18].

The digital age is upon us and is, to varying degrees, integrated into everyday life in most countries around the world. In Sweden, 92% of the population has a computer, 93% has access to the internet, 56% owns a tablet, and 77% owns a smartphone. Most families with children (87%) have multiple computers, tablets, and smartphones. The age at which children start using the internet is notably earlier nowadays (67% of 3-year olds), and the proportion of children using it daily increases with age (32% at the of age 2 years, 50% at 6 years, 75% at 10 years, and 96% in teenage) [19]. The use of Web-based technology to prepare children for pediatric care is increasing, and it provides almost unlimited opportunities for the development and design of such programs. However, preparation of children involves more than delivery of information. Receiving information does not mean one has learned and understood. Learning is a process of constructing one’s own understanding [20,21]. Children need to process information about their illness and health to learn about and fully understand their condition [22,23]. Focus on the design and development of preparation programs for children prior to contact with the health care system, therefore, has to change from only providing information to encompassing children’s learning processes.

Anaesthesia-Web [24] exemplifies a well-received and worldwide used website to prepare children for hospitalization, anesthesia, and surgery. Even though the development of Anaesthesia-Web was based on children’s experiences, a comprehensive pedagogical perspective on the website is lacking. In this study, we analyzed the content and design of Anaesthesia-Web based on a theoretical pedagogical framework. A deeper theoretical and pedagogical understanding encompassing children’s learning processes on Anaesthesia-Web may optimize and support the development and design of similar websites for children in pediatric care.

### The Development of Anaesthesia-Web

The content and design of Anaesthesia-Web was developed and produced by a multidisciplinary team of around 150 persons including health care professionals, computer programmers, Web designers and Web design students, journalists, authors, television producers, advertising agencies, and photographers recruited from children’s magazines and television shows. The adolescent parts of the website were created together with a popular Swedish author, and the design was both modern and stylish to suit the age group. The team also included parents and children aged 4-16 years with different ethnic backgrounds and experiences of hospitalization, anesthesia, and surgery. The aim of including people with different perspectives in the developmental process was to explore the need for preparation as well as to understand how the content could be best presented and understood among different groups of users. The multilingual work played a central role during the development of Anaesthesia-Web. Native speakers of all available languages on the website were included in the development team. Translations of all manuscripts were completed by authorized translators who were experienced in translating text from the medical context. All text was proofread by native speakers with medical knowledge who were also translating for the web programmers during the implementation of the text to the website. The recordings of all text involved around 25 native speaking actors per language in appropriate ages for all the characters.

### Previous Evaluations of Anaesthesia-Web

In order to understand the usage and distribution of website data on total numbers and geographical distribution of the visitors, the most visited parts of the website and visitor’s interactions on notice boards were registered continuously and analyzed descriptively over a period of 5 years (2009-2013). Visitors were registered through their internet protocol addresses. Search engines and websites with a ping back were also registered. All statistics were collated using a log analyzer, generating advanced Web, streaming, file transfer protocol, or mail server statistics graphically. Anaesthesia-Web had an average of 120,000 visitors from approximately 100 different countries annually. The number of visitors was equally distributed over the years, months of the year, and days of the week. Around 300 different websites link to Anaesthesia-Web. Most visitors find the website via the Web address (62,040/120,000, 51.7%) and search engines (50,760/120,000, 42.3%) and the rest via other websites (7200/120,000, 6%). The most common keyword combinations for finding Anaesthesia-Web on search engines were: *anaesthesia and children*, *anaesthesia and risks*, and *risks with anaesthesia*. Analysis of the “Top 5” most visited parts of Anaesthesia-Web during November each year between 2009 and 2013 showed that the most popular parts of the website have stayed quite stable during the years. The most frequently visited parts of Anaesthesia-Web were the “playful”parts and the written part of the website describing general information about anesthesia.

In a previously published randomized controlled trial, including 125 children and parents undergoing outpatient surgery, Anaesthesia-Web was compared with conventional printed brochure material. A set of 6 questions was assembled for children as well as for parents. A prerequisite was that a complete answer to the chosen questions should be available both in the Web-based option and the brochure material. All questions should be relevant to anesthesia. The primary endpoint was to compare the total question score of correctly answered questions by children prepared using the Anaesthesia-Web or conventional printed brochure material.

Secondary endpoints were the total question score for parents and the influence of age, gender, and time between the preoperative visit and day of surgery. The main conclusion was that Web-based interactive preoperative preparation results in higher total question scores in children aged 3-12 years and in their parents compared with conventional brochure material [25].

### Objective

The objective of this study was to elucidate key educational principles in the development and design of websites for children in pediatric care.

## Methods

### Research Approach

A directed qualitative content analysis [26] was applied to illuminate and explain prerequisites for children’s learning on a website preparing children for hospitalization. The chosen approach of content analysis is signified by applying predetermined variables or concepts to interpret a text or content. A directed qualitative content analysis is used when existing theoretical or empirical knowledge about a subject is judged to enhance the understanding of a certain research question. The aim is to describe the common themes characterizing the object being studied. In this case, the design and the coherent content of Anaesthesia-Web constituted the data being analyzed. The predetermined concepts applied in this analysis were derived from a theoretical pedagogical framework.

### Anaesthesia-Web: Content of the Analysis

**Figure 1:**
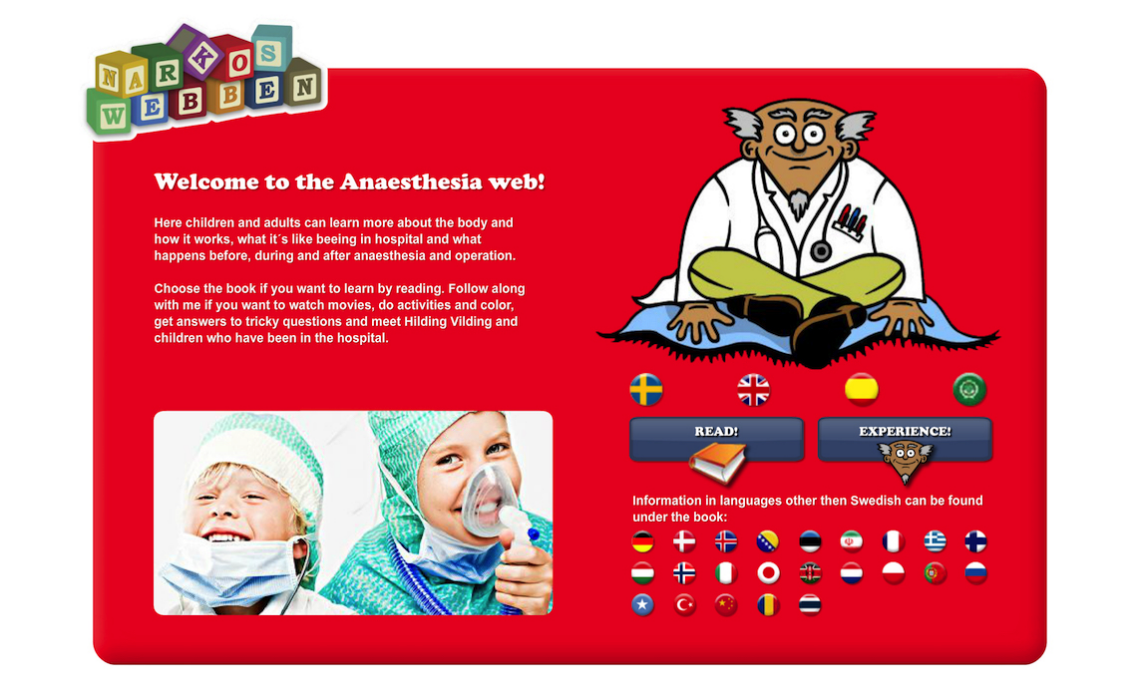
The front page of the Anaesthesia-Web website. Copyright: Anaesthesia-Web.

Anaesthesia-Web (Figure 1) represents a comprehensive, interactive, age-appropriate, multimedia, Web-based portal to prepare and educate children and families prior to contact with the health care system. On Anaesthesia-Web, children can learn about the body and how it works; what it is like to be hospitalized; and what happens before, during, and after anesthesia and surgery.

### The Content and Design of Anaesthesia-Web

**Figure 2:**
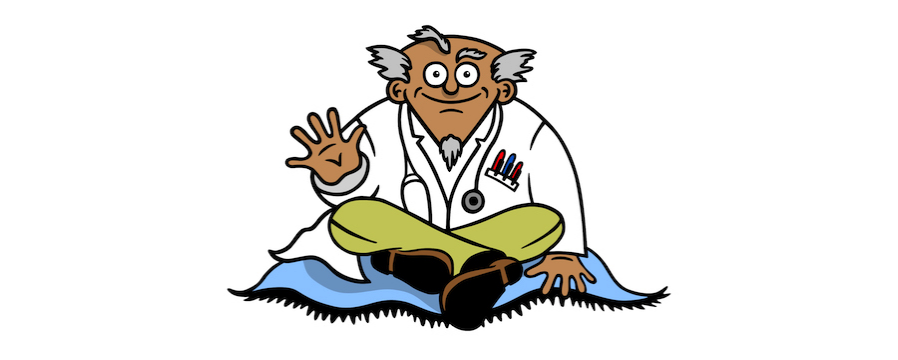
Doctor Safeweb is available all over the Anaesthesia-Web to guide visitors and to answer frequently asked questions. Copyright: Tintin Timén and Stefan Wahlberg.

**Figure 3:**
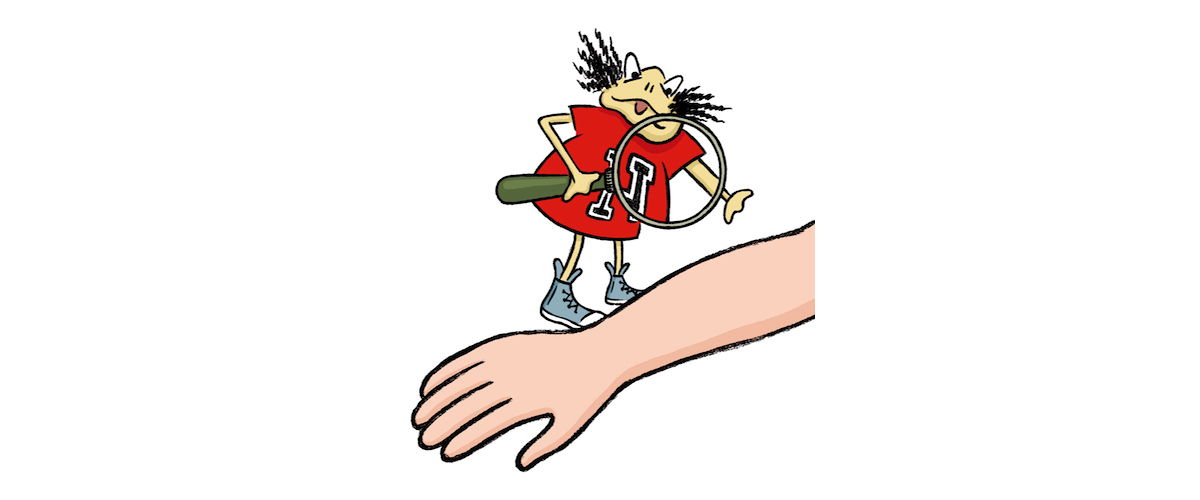
The curious spy-scout Hilding Vilding helps visitors to investigate the hospital on the Anaesthesia-Web. Copyright: Tintin Timén and Stefan Wahlberg.

**Figure 4:**
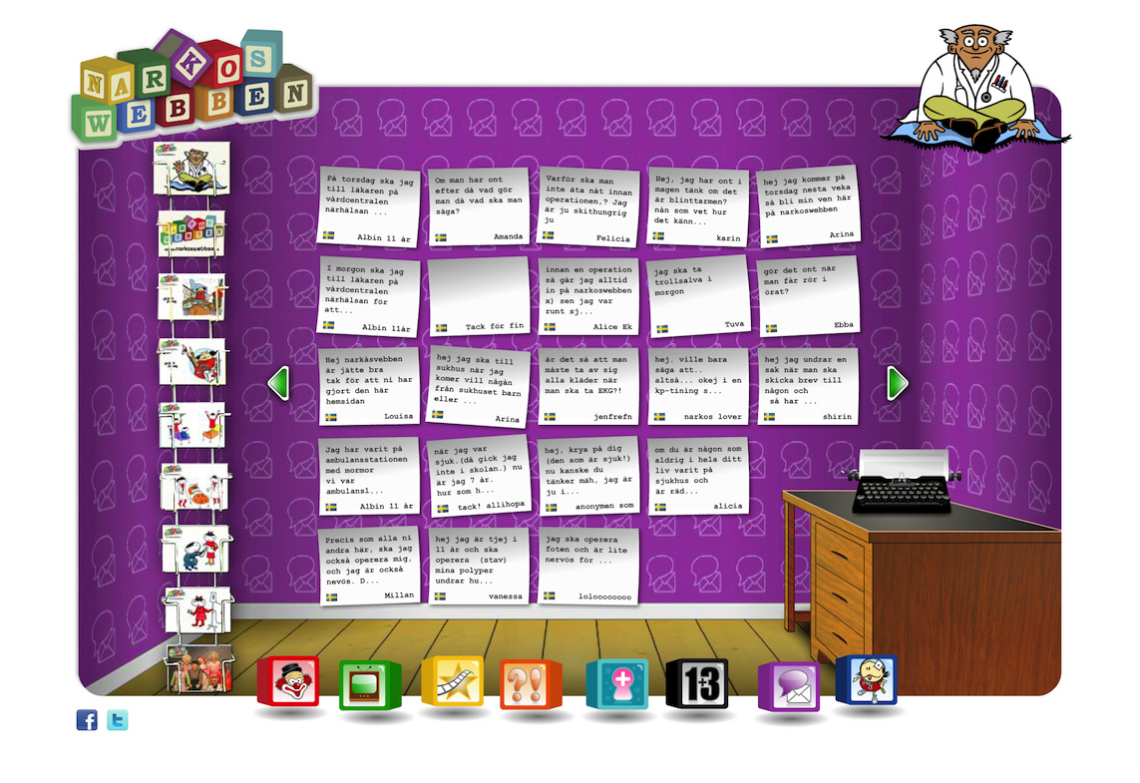
The Anaesthesia-Web has notice boards where children can ask each other questions and share experiences in text and drawings. Copyright: Anaesthesia-Web.

The content of the information provided on Anaesthesia-Web is based on evidence and clinical experience from different contexts including medicine, children’s cognitive developmental science, and Web-based technology. Common concerns from children and parents before hospitalization include uncertainty and outcome of procedures, separation, loss of control, needle sticks, pain, and risks associated with anesthesia and surgery [27-30]. The website aims to provide learning possibilities as preparation for these scenarios with information for toddlers (1-3 years), pre-school children (3-5 years), school children (5-12 years), adolescents (12-18 years), and parents. Anaesthesia-Web comes in two different parts, which map on to the traditional “For Children” and “For Adults” distinction, but are in practice labeled: “Read” and “Experience.” Anaesthesia-Web contains a wide range of communication modalities such as films, cartoons, Web books, games, blogs, videos, and interviews with children of different ages. Two characters, Doctor Safeweb (Figure 2) and Hilding Vilding (Figure 3), are key features of Anaesthesia-Web. Doctor Safeweb is available all over the website to guide visitors and to answer frequently asked questions. He conveys all information in both writing and with recorded narration. Hilding Vilding works as a curious spy scout in the hospital. He is as tiny as the palm of the hand, which means he can be present everywhere and investigate everything without being discovered. Two notice boards are available on Anaesthesia-Web, one for younger children and one for adolescents. On the notice boards, children can ask each other questions and share experiences using text and drawings (Figure 4).

The information on Anaesthesia-Web is generally applicable, which means that the website can be used regardless of the health care setting to which the family presents. Anaesthesia-Web is available in Swedish and 3 major world languages (English, Arabic, and Spanish) and contains written information in 27 languages. Anaesthesia-Web has open access with different URL addresses.

### Directed Theoretical Content Analysis

The theoretical framework is based on a combination of learning theories and especially considered in relation to research on children’s learning. Please see the Multimedia Appendix 1 for further explanation. In the theoretical pedagogical framework, learning is regarded as an active construction process and an individual’s life-world is the basis for his or her understanding, thinking, and action [20,22]. Learning involves the whole person and is defined as a meaning-making-construction process about new or modified interpretations of perceptions and experiences [20,21]. The educational concepts of *preunderstanding*, *motivation*, *learning processes*, and *learning outcome* (Multimedia Appendix 1) were used to analyze the learning possibilities with Anaesthesia-Web for children prior to contact with pediatric care.

#### Preunderstanding

Preunderstanding is a significant part of learning built on emotional, cognitive, and practical live experiences; knowledge acquisition; and reflections, which are more or less conscious. Preunderstanding is a prerequisite, and constitutes the basis, for the interpretation of new experiences and thoughts and for understanding and appraisal of what is seen, heard, and experienced [20,31,32]. The individual interpretation of the world always starts with what is already known, which helps to not only understand but also react if something seems odd, different, or frightening. Although there is awareness of preunderstanding, it is often not apparent that it will direct individual attention and action. Preunderstanding can thereby be a barrier for learning when thinking is obstructed and the ability to see and consider other perspectives decreases [22].

#### Motivation

Motivation to learn is vital to stimulate the start and maintenance of a learning process [22,32-34]. Motivation can be triggered not only by the experiences of something being fun and exciting [35] and by internal and external factors but also when previously used approaches to solve problems are not working and when new questions arise that need to be answered and investigated [22,32-34]. Motivation is stimulated both by the challenge and experience of having to master something, as well as by the feeling of succeeding [36].

#### Learning Processes

An individual’s processing of information is central and constitutes the essence of the learning process. A learner not only receives information but also interprets and connects it to the existing knowledge, thereby constructing new understanding. Feedback on learning achievements is very important in the learning process [37,38]. All senses are needed to capture new information and to process the existing knowledge cognitively, emotionally, and by active participation. By processing new information and analyzing the old and new understanding, new understanding and knowledge can be developed [20,31].

#### Learning Outcome

Learning processes are meant to result in understanding, ability to perform skills, and, maybe, changed attitudes and behaviors depending on the learning situation [20,22,39]. In this case, the learning goals are related to children and parents being prepared for hospitalization and more specifically for anesthesia and surgery. This means for the child to understand what is going to happen and be able to cope with the situation. Moreover, it is important that both children and parents experience safety and confidence. Feedback on learning achievements is important to support the learner to be confident that the message is understood correctly or to clarify that the information should be repeated for improved understanding [38,40].

In the first phase of the analysis, the predetermined concepts to be applied on Anaesthesia-Web were chosen and described according to the basic theoretical pedagogical framework. In the second phase, the learning concepts were systematically applied on Anaesthesia-Web to identify salient learning opportunities such as how to get access to information, different kinds of multimedia, and possibilities for interaction and guidance. In the third phase, the salient learning opportunities were analyzed using a combination of learning concepts and knowledge about children’s learning in the context of health care and especially relating the analysis to the features of Web-based learning. This iterative, analytic process, based on the theoretical pedagogical framework, helped identify themes that mirrored children’s opportunities to learn on a website prior to a hospitalization.

The research group comprised different perspectives including Web-based learning, medical education, technology-enhanced learning, pediatrics, and anaesthesia. Two researchers (GL and CS) performed the initial analysis, and the whole group negotiated and agreed on the results to ensure trustworthiness [41,42].

## Results

### Themes

In the analysis of Anaesthesia-Web related to the central learning concepts *preunderstanding*, *motivation*, *learning processes*, and *learning outcome* (see Multimedia Appendix 1) we found 4 themes related to children’s learning: *In charge of my learning*; *Discover and play*; *Recognize and identify*; and *Getting feedback*. The correspondence between the concepts and the themes is presented in Table 1.

### Theme 1: *In Charge of My Own Learning*

This theme involves the central learning concepts *preunderstanding* and *motivation*. Based on their level of knowledge, interest, and interpretation, children themselves can decide where to start and how to use Anaesthesia-Web. This allows them to be in charge of their own learning, which is an important motivational factor [22,32-34]. Instead of classifying children as one equal group, Anaesthesia-Web acknowledges children as a diverse group in which the need and format for information differ. All information on Anaesthesia-Web is adapted to children’s different cognitive and developmental stages. This includes, for example, the vocabulary, the length of stories and films, and the configuration and design of characters and their expressions. However, there are no signs connected to age on Anaesthesia-Web, and thus, it is up to everyone to choose what and how to use the content provided.

Children’s past experiences of sickness and health care vary, and Anaesthesia-Web enables children to put their previous experiences into a new frame of reference and enhance their thinking and learning. Anaesthesia-Web contains a wide range of multimedia such as films, cartoons, Web books, games, blogs, videos, and interviews with children of different ages. Here, children are able to take part in a hospital adventure together with the hospital’s clowns, potter around and paint, create their own operating theater, watch a film, and meet children with different experiences of hospitalization. On the notice boards, children can ask each other questions and express and share experiences in texts, drawings, paintings, or photos. In addition, there is information on different forms of anesthesia, sedation, pain alleviation, and answers to frequently asked questions from children and adults. Parents receive suggestions on how to prepare both themselves and their children prior to hospitalization.

**Figure 5:**
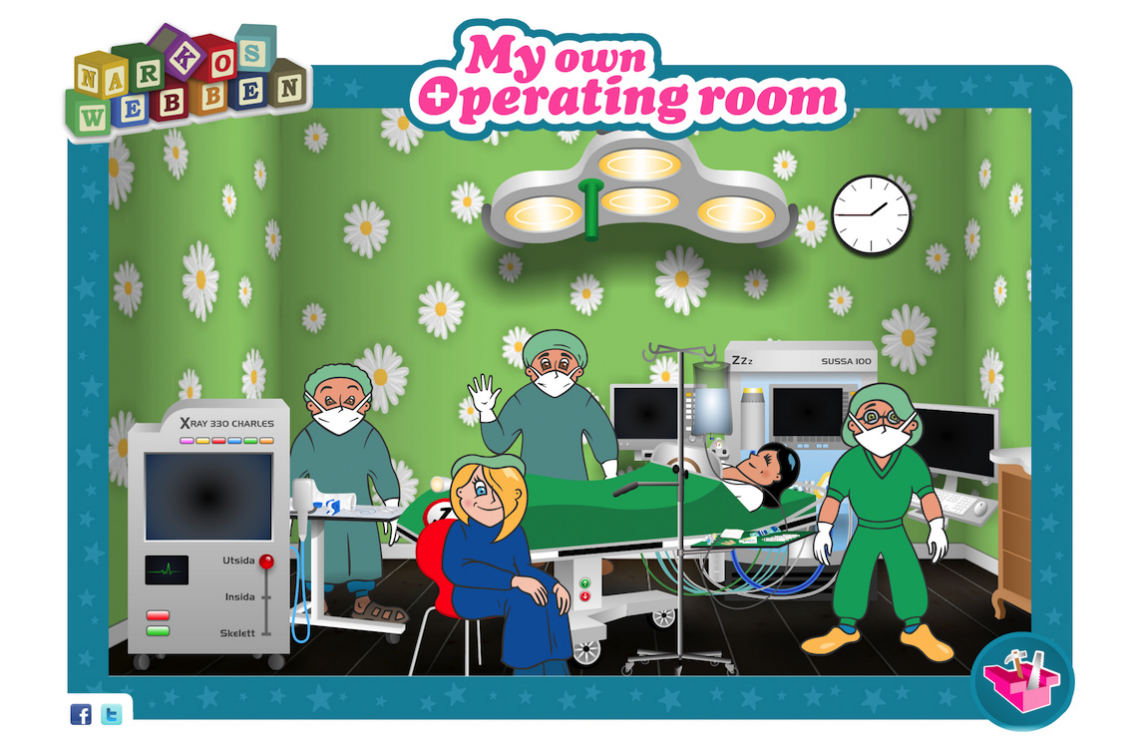
The Anaesthesia-Web's "My own operating room" gives children the opportunity to play and explore what it is like to be in an authentic hospital situation. Copyright: Anaesthesia-Web.

Anaesthesia-Web can extend children’s ability to learn by enabling exposure to ideas and experiences that otherwise would be inaccessible. In “My own Operating Room” (Figure 5), children can construct their own reality by taking command and choosing what procedures they want to experience and what professions they want to play. Maybe they want to change roles being nurses or doctors. Anaesthesia-Web provides children with tools to imagine and explore what it is like to be in authentic situations. They get the opportunity to experience roles in a real-life setting and, at the same time, learn about the setting itself.

Doctor Safeweb and Hilding Vilding, the central characters on Anaesthesia-Web, support the children to take charge of their own learning. By conveying all content in both writing and with recorded narration, children with special needs, hearing and visual impairments, and reading difficulties are given equal access to preparation and learning. For immigrant children, all information on Anaesthesia-Web is available in Swedish and 3 major world languages (English, Spanish and Arabic). Doctor Safeweb, Hilding Vilding, all characters, and animated animals are fluent in these languages.

### Theme 2: *Discover and Play*

This theme involves the central learning concepts *motivation* and *learning process*. The content on Anaesthesia-Web is mediated by playful interactive elements to stimulate children’s natural curiosity, knowledge seeking, and motivation, factors which are all crucial to initiate and maintain the learning process. Children can use play to seek new knowledge and make events possible to understand. The interactive parts of Anaesthesia-Web enable children to not only prepare for upcoming events but also process what has happened. On Anaesthesia-Web, children can learn and experience how to give an injection, how to bandage a wound, and plaster a broken leg. They can monitor the heartbeat and measure the blood pressure.

**Table 1:** Themes related to learning concepts on Anaesthesia-Web.

Learning concepts	Themes			
	In charge of my own learning	Discover and play	Recognize and identify	Getting feedback
Preunderstanding	✓		✓	
Motivation	✓	✓	✓	✓
Learning Process		✓	✓	
Learning Outcome				✓

**Figure 6:**
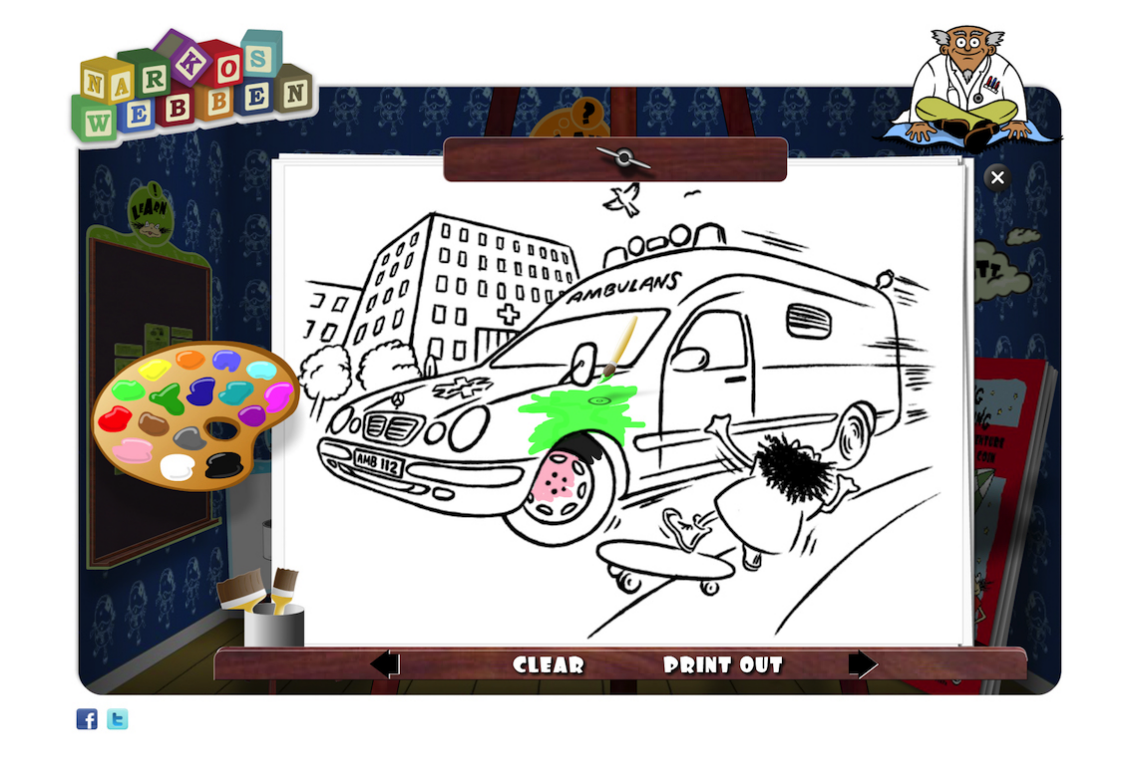
Children can play and learn from Hilding Vilding's coloring and craft book on the Anaesthesia-Web. Copyright: Anaesthesia-Web.

In “My own Operating Room,” children can decorate and furnish an operating room to their taste. They might want the operating room to have flowered walls and grass on the floor, with a pink operating table with comfortable pillows or a table that looks like a space rocket. They can also try various technical functions such as using different monitors or operating the suction, surgical lights, and tables. Anaesthesia-Web’s main characters Doctor Safeweb and Hilding Vilding play significant roles in stimulating children’s motivation for learning. Hilding Vilding is filled with questions and does not stop asking them until he has found the answers. With the answers on hand, Hilding Vilding is a master at explaining difficult and complex things in an easy and understandable way. Children can follow Hilding Vilding through an exciting adventure inside the body, playing and learning from his coloring and craft book (Figure 6).

At Anaesthesia-Web, children can design and create their own bandage, email it to a friend, or print and frame it. When playing the “X-ray” game or the “body memory game,” they can learn about the body and how it works. In the “Intravenous game,” they can give injections and start intravenous infusions. In the “Pain quiz,” children can learn how to estimate the level of pain as well as strategies to cope with pain. Multimedia formatting offers choices of interfaces (text, images, sounds, and animations) as the use of all senses for processing and interpreting information is known to be beneficial for children’s learning.

### Theme 3: *Recognize and Identify*

This theme involves the central learning concepts *preunderstanding*, *motivation*, and *learning process*. The learning process depends on a will to be engaged, interested, and experience the effort as meaningful. Children’s preunderstanding can stimulate their motivation to learn about what is going to happen to them in the hospital, but the Web-based information can also be a hindrance if the information is frightening or if children do not perceive that it is directed toward them. Recognition and identification are the important factors for children to experience the visit as meaningful and to maintain the motivation to learn.

**Figure 7:**
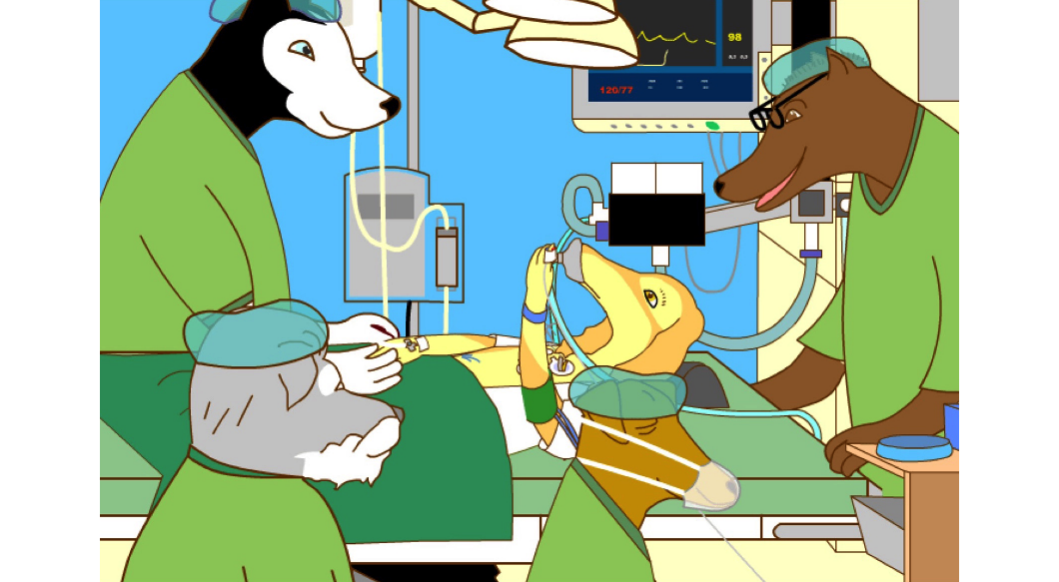
Neutral and de-identified characters undergoing examinations and treatments on the Anaesthesia-Web. Copyright: Anaesthesia-Web.

The diversity of characters available on Anaesthesia-Web offers children with different backgrounds the possibility to find someone with similar experiences they can recognize and identify with. The content on Anaesthesia-Web is nontime sensitive and without time-dependent factors such as hairstyles, clothing, and accessories. Characters and their appearance are neutralized and deidentified. A number of characters consist of sick animals and teddy bears undergoing examination and treatments (Figure 7). Toddlers can identify with cuddly toys, and the information for school children is adapted for this age group’s curiosity. Adolescents can gain information from others who have blogged about their experiences while hospitalized.

**Figure 8:**
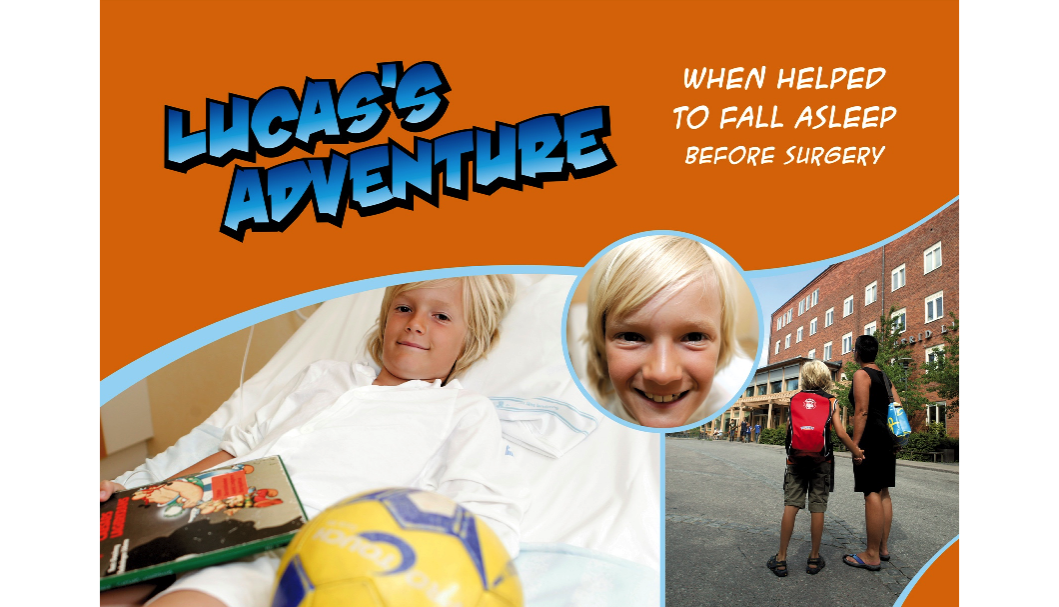
In the web-magazine “Luca's adventure”, children are gradually introduced to anaesthesia and surgery by following someone experiencing the same procedures as they will. Copyright: Anaesthesia-Web.

Anaesthesia-Web does not contain any hospital-specific or procedure-specific information, and all interiors are created with generalizable features to help children identify with the information regardless of where and why the child presents for health care. Hilding Vilding plays a key role in fostering this: he is afraid but, at the same time, is curious to explore the hospital, has a lot of questions, and wants to understand and learn. He gets answers to almost all questions, including the ones children probably would never dare to ask. Since Hilding Vilding always makes himself exhausted by asking even the dumbest questions, he allows children to feel that they are always doing better than himself. Hilding Vilding confirms that it is natural to be afraid and clarifies that being curious, asking questions, and searching for answers is the only way to learn something new and that when you have learned something new, you often become a little less frightened. In the Web-based magazine “Lucas’s adventure,” (Figure 8) children are gradually introduced to steps associated with anesthesia and surgery. By following someone who experiences the same procedures as they themselves will, children are given the opportunity to recognize situations and gain insight and understanding in advance.

The notice boards on Anaesthesia-Web help recognize and identify others in the same situation. Children focus on their own fears and experiences associated with different medical conditions, hospitalization, anesthesia, and surgery and those of their siblings and friends. They discuss symptoms; treatments; and side effects, especially their fear of needles, injections, and painful procedures. Fasting routines before and after anesthesia and preoperative and discharge procedures are also commonly discussed. On the notice boards for adolescents, discussions are most often about fear of exposing themselves during examinations and treatments and anxiety about losing consciousness and control.

### Theme 4: *Getting Feedback*

This theme involves the central learning concepts *motivation* and *learning outcome*. From an educational perspective, feedback is crucial in giving the visitors the opportunity to not only test their level of knowledge but also reduce fear and generate trust and confidence. On Anaesthesia-Web, children get immediate feedback on their performance and progress without any delay, which increases motivation and concentration and retains attention. Doctor Safeweb has a central role in giving advice and feedback when children explore Anaesthesia-Web. He is available all over the website to guide and to give confirmation, feedback, and answers to frequently asked questions. By getting an immediate feedback on the failure of an idea, children have a chance to correct, learn from errors, improve performance, and achieve goals. On the notice boards, children can participate in discussions and get feedback to questions from peers facing similar experiences. On a website dealing with sickness and hospitalization, feedback that promotes trust and confidence is vital. In this, Doctor Safeweb has a warm, secure, and faithful personality that encourages children and parents to maintain their motivation for learning when encountering new and sometimes frightening situations.

## Discussion

### Principal Findings

Web-based information can be interactive and patient centered, but if it is not used with the consideration of children’s learning processes, it might work only as another source of information. In this study, the content and design of Anaesthesia-Web were analyzed from an educational perspective. The concepts of *preunderstanding, motivation, learning processes,* and *learning outcome* were used to analyze the possibilities for children to learn on Anaesthesia-Web prior to contact with health care system. In the analysis of Anaesthesia-Web related to central learning concepts, we found 4 themes: *In charge of my learning*; *Discover and play*; *Recognize and identify*; and *Getting feedback*.

Studies have shown that Web-based activities can be effective for reasoning, problem solving, and recognition of words, concepts, and situations at an earlier age than expected [43-46]. Therefore, the multimedia diversity in combination with the visitor’s freedom on the website is of importance to stimulate children’s learning based on their varied background, knowledge, abilities, and what they find as meaningful. Children need opportunities to learn in ways that work for them [28,45,47,48]. Preunderstanding will direct children’s attention, which might be helpful when navigating on the website; however, it can also become an obstacle to learning. This complexity is important when designing a website for children within the health care context. For many children, the information on the website is their first meeting with the health care system, whereas others have a lot of experiences, which unfortunately are not always positive. Children with previous experience of hospitalization are not protected from fear. On the contrary, their concerns and anxiety are often increased because they know what to expect and because previous approaches to solve problems and answer questions may have failed [17,28]. Therefore, when designing Web-based learning opportunities, it is crucial to consider this group of children. With increasing cultural diversity and global mobility, it is important to be aware that hospitalization can be a very traumatic experience for migrant children; language, cultural and religious beliefs, and previous experiences of health care and hospitalization in these children demand specific prerequisites for preparation and learning [49]. By creating opportunities for migrant children to be in charge of their learning in their native language, the risk of unnecessary anxiety as well as misunderstandings will decrease.

Our analysis shows that Anaesthesia-Web provides crucial prerequisites for any visitor to take charge of their learning. The content is adapted to children with different experiences, backgrounds, ages, knowledge, culture, developmental stages, and abilities, aiming to provide information suitable for everyone. This is in line with educational research, which shows the importance of offering opportunities for meaningful learning [22]. The content is presented and designed to provide different kinds of learning opportunities, offering multimedia diversity and ease of access for the visitor to make individual choices. By designing multiple approaches to solve problems, answer questions, and investigate information, meaningfulness and motivation to learn can be triggered [35].

Research into children’s learning with Web-based technology in schools has shown that computer programs offer children some control over learning activities and provide opportunities for choices or imaginative expressions, facilitate children’s creative approaches to learning, and increase interest and engagement [50]. Children will need guidance and support to get interested and make choices because it is a great challenge for children to approach the frightening situation associated with preparation for a hospitalization. On Anaesthesia-Web, this is managed by the two central characters: “Doctor Safeweb,” representing order and safety, and “Hilding Vilding,” introducing fun and curiosity, initiating challenges, and confirming that it is possible to take different routes to learning and discovery.

The theme *Discover and play* represents the core of the content and layout on the website. Discovery and play are interrelated, but the concept “discover” contains important additional features for learning. Exploration and play are well documented as important factors in children’s learning, and the theme highlights significant educational factors connected to the stimulation of motivation and processes involved in learning. The website helps children explore the hospital environment and what is going to happen to them while in hospital. The content and design are developed to stimulate and motivate children’s curiosity, creativity, engagement, incidental learning, and active participation, and they can approach the situation playfully, asking questions and finding answers. Playing may reduce the pressure associated with achievements or need to learn [51], providing children with a minimum of risks for experiences related to mistakes and inadequacy in their preparation for hospitalization. Children’s motivation has been shown to increase when they are involved with engaging and fun Web-based technology [50]. Computer learning activities can elicit high levels of interest in and focus on a learning task that does not tend to diminish over time [45,52]. These studies relate to learning in school, but it seems likely that this knowledge is applicable to our target population. On Anaesthesia-Web, visitors are given possibilities to prepare for and process hospitalization by accessing information; by practicing skills, functions, and procedures; and by experimenting with different roles in a real-life hospital setting while learning about the setting itself. Children, therefore, receive, process, and apply information cognitively, emotionally, and by active participation. By processing new information and analyzing the old, new understanding and knowledge can be constructed [20,31,45]. As a tool in the learning process, the computer gives the learner specific opportunities for information seeking, communication, and processing of information [50,51,53]. The use of visualization, modeling, and simulation have been proved to be powerful tools to increase children’s understanding of scientific concepts and underlying phenomena [45]. By providing children with tools to help them understand and manage procedures, they may be able to transfer what they experience on the website to the real-world context [51]. This is extremely important when designing a website to prepare for a real event [45,50,53-55]. It has also been shown that Web-based technology is beneficial to engage children in collaborative learning, reasoning, and problem-solving activities that had been thought to be too sophisticated for them to understand and perform at very young ages [46].

The third theme *Recognize and identify* is crucial when preparing children for contact with the health care system. To find it meaningful to enter and use the website, children first need to recognize and identify with the content and characters. Second, they need to recognize and identify themselves as persons needing to learn and prepare prior to a hospitalization. Building on children’s view of their own thoughts, concerns, and experiences of sickness and hospitalization has been shown to be essential in the development of Web-based preparation programs in health care settings [56,57]. When developing Anaesthesia-Web, a panel of 15 children, aged 4-16 years, with different experiences of sickness and health care were involved. The development of the content together with the target group is important for children’s need to not only identify with others facing similar situations [48] but also increase the acceptance and use of the information provided. On Anaesthesia-Web, children can interact with a diversity of characters and find someone to identify with. Accompanied by the safe and trustful character Doctor Safeweb, children are guided and supported to explore step-by-step the strange and maybe frightening situations at the hospital. The fantasy character Hilding Vilding acknowledges the feelings of fear and worry to help overcome barriers for learning. The presence of notice boards on the website helps identifying with others facing the same situation. A child’s identity is enhanced by participating in a community or becoming member of a group [58] and can be a powerful motivator for learning. Identification with others increases interest and engagement, enhances meaning, and results in an increased motivation to learn.

The fourth theme *Getting feedback* highlights the possibilities to verify and confirm that the learner has managed, understood, made progress, and received acknowledgment for achievements and performances. Feedback is crucial for keeping up the motivation to learn and is necessary to enable the judgment of what has been learned. The best forms of feedback supporting learning involve interactive processes [37,38]. This is a challenge to accomplish on a website concerning preparation for hospitalization accessed in advance at home. Features promoting feedback on Anaesthesia-Web include quiz games, answers to frequently asked questions, and performance feedback for practical skills with guidance by Doctor Safeweb. The notice board offers the possibility to discuss, share experiences, receive feedback, and learn from others facing similar situations. Studies of children’s learning using Web-based technology have indicated that learning proceeds most rapidly when learners are provided with different levels of challenge, when they have frequent opportunities to apply the ideas they encounter, and when feedback on the success and failure is received immediately [45]. When designing games, it is important to ensure that the game structure suits the learning objectives. Children seem to like unpredictability, audio effects, and games with scoring opportunities where the speed of an answer counts [51]. An improvement suggested by our analysis could be for children to have the possibility to chat and receive immediate feedback on questions and concerns from the hospital.

### Implications for Designing Health Care-Related Websites for Children

By developing preparation programs based on pedagogical knowledge and experience of children’s learning processes, we believe that Web-based technology can be used to its fullest advantage as a health care learning resource. The themes found in the analysis of Anaesthesia-Web provide a basic structure that captures the key educational features needed to prepare children for contact with health care system. Communication with health professionals is an area for further development, but opportunities for children to communicate with others facing similar health challenges and experiences is an important advancement [50]. Learning using Web-based technology is most effective when there is active engagement, participation in groups, frequent interactions, feedback, and connections to the real world [45]. Identification with others creates interest and engagement, which, in turn, lead to meaningfulness and an increased motivation to learn about one’s own situation. Web-based technology can also be a solution for children with special needs for social interaction, communication, and learning [43,59-61], allowing them to participate in reality-based activities that would otherwise not be possible for them [62]. According to social learning theories, certain behaviors can be learned and reproduced, under similar conditions, by observing the actions performed by others [63].

The abovementioned research about improvements in children’s problem-solving abilities as well as abilities to abstract and engage in reflective thinking using Web-based learning activities is well worth looking into to increase the learning opportunities for children prior to a hospitalization [45,46,50,51]. The development of sophisticated computer games has resulted in new approaches to learning principles, emphasizing the role of elaboration, playing, and engagement [51,64]. Through interactive learning using games, pictures, and sounds, children receive several associations that help them remember and assimilate new information [62].

For adolescents, the internet has become an important, valued, and frequently accessed information source for a range of sensitive health issues [2]. When designing prerequisites for adolescent’s learning, it is of highest importance to consider how to meet this group’s preunderstanding by providing the information at an appropriate level, balancing between childhood and the adult world. It is a challenge to develop and iteratively refine systems that are attractive enough to catch children’s and young people’s interest, are useful, and keep the visitor engaged to the website [56,57,65,66]. We would argue that a website has only one chance to catch a visitor’s attention, and therefore, it is important to carefully consider how to develop the content and design to be serious and trustworthy as well as secure an active updating [56]. The phenomenon of attrition applies to a varying extent to most eHealth interventions [67]. Active updating is an important task that can be seen as the continued existence of a website in terms of keeping visitors interested through continuous adjustments to maintain presence, interest, and the website as a living tool [56]. Another factor to be aware of is that children of all ages are extensive media consumers, which may have resulted in a distorted picture of sickness and hospitalization. Providing children with reality-based information is, therefore, important to help them regulate their expectations and allay their fears [56,68].

### Methodological Limitations

The choice of learning theories and the assumptions about learning they mirror has influenced the analysis and the result. To ensure credibility and make it possible for researchers and readers of the paper to transfer the results to other contexts, the theoretical perspectives on learning were described in detail (see Multimedia Appendix 1) and were applied systematically [41] by an experienced multidisciplinary group.

## Appendix

Multimedia Appendix 1 Supplementary Appendix.
