# Supplementary material for: Web-Based Learning for Children in Pediatric Care: Qualitative Study Assessing Educational Challenges
Source: JMIR Perioper Med. 2018 Sep 25;1(2):e10203. doi: 10.2196/10203 (PMC7709848; doi:10.2196/10203)
Supplement: Multimedia Appendix 1 [file periop_v1i2e10203_app1.pdf]

Supplementary Appendix

Learning concepts

Learning is seen as an active construction process and the individual´s life world is the basis for the individual´s understanding, thinking and action [1, 2]. Learning is a meaning making construction process about new or modified interpretations of perceptions and experiences and involves the whole person. Meaning, knowledge and understanding are created based on different kinds of information, data and by and in interaction with people, artifacts and the environment [2, 3]. No one can passively receive understanding and skills from others. Learning is a multifaceted phenomenon which requires processing of information cognitively, emotionally, socially and through testing and practical actions [4, 5]. Meaningful learning in the sense of understanding and comprehension consist of a cognitive and a social dimensions, that interacts in the learning process [6, 7]. Bandura [6] and Vygotsky [7] emphasize that learning is social in nature. We learn and construct meaning from and in interaction with others and the environment. Already Dewey [8] described that awareness and understanding of what we encounter and experience is dependent on continuous stimuli from other people and the environment. The individual takes on values and desires of others and assesses successes and failures in relation to others and the social context. Web-based technology opens up possibilities and offers approaches to learning in congruence with the assumptions about learning mentioned above [9-12]. Interactive play constitutes a positive driving force for learning without constraints and compulsion and enables children to be active and with the use of all senses explore new ways of understanding for learning [9, 12, 13]. Compared to "traditional education materials" web-based technology can expand the range of things that children can create and in doing so enable them to encounter ideas that were previously, without the new technology, not accessible to them [10].

As a learning tool web-based technology offer a number of advantages also in the health care context, including the availability, tailoring of information for the individual needs, a private learning environment and an immediate reinforcement of the learning that has occurred [14-18]. Web-based technology also enables contact with experts or others facing similar health challenges. The social integration and sharing of information that occurs through these connections may increase patient´s involvement, learning and understanding of their medical conditions [18]. New communicative conditions make learners become not only consumers of information but also producers of information and active members of learning communities. With new resources for communication, new demands and new possibilities are raised for learning [19].

Pre-understanding

Pre-understanding, described by several researchers [2, 4, 8] as significant for learning, means all humans has conceptions and perceptions about situations we are facing. Pre-understanding is built on emotional, cognitive and practical lived experiences, knowledge acquisition and reflections which are more or less conscious. Pre-understanding is a prerequisite, and constitutes the basis, for interpretations of new experiences and thoughts, understanding and appraisal of what is seen, heard

and experienced. The individual interpretation of the world always starts with what is already know which helps to understand but also to react if something seems odd, different or frightening. Although the awareness of the pre-understanding it is often not apparent it will direct the individual attention and action. Pre-understanding can thereby be a barrier for learning when thinking gets obstructed and the ability to see and consider other perspectives decreases. Children bring varied levels of experience and learning preferences to the educational environment that is offered. From a pedagogical perspective it is a challenge to understand existing features of the pre-understanding of a group of children, like children of a certain age, as well as the variety of children's individual pre-understanding within a group. Pre-understanding of children in the same age will vary depending on their previous experiences, knowledge and approaches to learning. Careful consideration needs to be given to what sort of information children should receive and when and how it should be provided [16].

Motivation

Consensus can be found among pedagogical researchers that the learner´s motivation is vital to stimulate the start and maintenance of a learning process. Some common features related to the characteristics of motivation have been highlighted in different learning theories [1, 8, 20, 21]. The experiences of meaningfulness are crucial to stimulate the motivation to learn. The learner has to be driven by a will to understand and/or manage something. To learn has to be important, out of different reasons. Meaningfulness can be triggered both by external factors, like "I will get a reward of some kind" or "someone will be very proud if I manage something", or internal factors, like "my curiosity is awakening" and "I want to find out how something works". Meaningfulness is also triggered when previously approaches used to solve problems are not working and new questions needed to be answered and investigated arise. The individual experiences an urgent need to understand and begins to search for information of different kinds in order to cope with the situation. The experiences of something being fun and exciting are also important for meaningfulness [22]. According to Piaget [20] humans pursue equilibrium in relation to the environment. Each action requires an interpretation of what we see and experience (assimilation). Insufficient understanding creates imbalance and the searching for explanations via reconstruction of thoughts, searching for explanations and understanding, to restore the balance (accommodation), starts. Achieving balance, to understand and manage, becomes an important form of feedback which, in turn, stimulates continued learning. Motivation is stimulated both by a challenge and the experience of having to master something, as well as by the feeling of succeeding [7]. Feedback on the learning achievements has turned out to play an important role in stimulating motivation and is also part of experiencing meaningfulness [23, 24].

Learning processes

The individual's processing of information in different ways and on different levels is central and constitutes the essence of the learning processes. The learner does not only receive information but also interprets and connects the information to already existing knowledge and thereby constructs new understanding. Feedback on learning achievements is very important this learning process [23, 24]. Knowledge is stored, interpreted and incorporated in the memory, in the brain using concepts related

in sematic networks. In order to recognize situations, facts and solve problems, new knowledge has to be associated with the individual existing conceptual structure. New information must have a new meaning for the individual to be included and perceived as part of the whole. This highlights the importance for the learner to process perceived problems and questions and not only be offered a complete answer [25]. A creative learning process can be based on an investigative approach to the situations and problems encountered by the learner. All senses are needed to capture new information and processing existing knowledge cognitively, emotionally and by action. By processing the new information, analyzing the old and new understanding, new understanding and knowledge can be constructed [2, 4]. Play constitutes a central part in children's life and an important part of their learning process [19]. Interactivity is an important part of children's play which enables for children to learn by using all their senses to understand the situations encountered [13]. Buytendijk defined play as an activity that is not oriented towards a specific goal; but a phatic way of perceiving the world where "the player" has an inner urge to move [26]. The concept "play", which relates both to "free" and rule-based activities, has connotations of many different kinds of activities and meanings for both children and adults. Play, playfulness and imagination can, in an overall perspective, be understood as a process of engagement, transformations of signs meaning making, reflections and meta-reflection. This perspective relates play to learning activities. Learning involves playing activities and playing can also be understood as learning activities [19]. Children's learning processes differ related to their age and cognitive development level, but also individually, like how much information they can process and how long they can keep up their attention.

The outcome of learning

Learning processes are meant to result in understanding, ability to perform skills and maybe changed attitudes and behavior depending on the learning situation [1, 2, 25]. In this case the learning goals are related to children and parents being prepared for a hospitalisation and more specifically for anaesthesia and surgery. This means for the child to understand what is going to happen and being able to cope with the situation. Of importance is also that both children and parents experience safety and confidence. The outcome of children's learning on a web-site will appear mainly when they attend the hospital, which may be too late. Thus, it is important to support children´s learning processes by enable optimal prerequisites for them to evaluate their learning via the web-site prior to the hospitalisation. Feedback on the learning achievements can support the learner to be confident that the message is understood correctly or make visible that one need to repeat or try again [23, 27]. The use of web-based technology has been shown to be associated with improvements in children´s development of concepts and cognition, knowledge and skills for thinking, planning, observing, problem-solving, creativity, reading, language, mathematics, hypothesis formation and testing. Well-designed web-based learning activities can improve skills of abstract thinking, reflective thinking, analyzing and evaluating information and scientific reasoning [9-12]. The dynamic nature of web-based technology seems to improve comprehension and help children to create mental models [28] and concretely explore abstract scientific concepts that would have been difficult to manipulate and learn without electronic components [9]. It helps them to understand health concepts and their

complex relationship and to formulate thoughtful and plausible theories about the events that occur behind the observable data [29]. Web-based technology has also been found to be effective for comprehension and recognition of unfamiliar words [12], understanding of cause and effect [30] and for introducing children to abstract concepts, that were previously considered too advanced for their age group [9]. Technology-based activities can also engage children in collaborative learning, reasoning and problem-solving activities that had been thought to be too sophisticated for them to understand and carry out at very young ages [12]. The fact that children are not only served the content but must be active stimulates creativity and imagination which leads to engagement and an extended attention span [9, 11, 31].

References

1. Illeris K. Contemporary theories of learning: learning theorists... in their own words: Routledge; 2009. ISBN: 1135226334.
2. Marton F. The experience of learning : implications for teaching and studying in higher education. Edinburgh: Scottish Academic Press; 1997. ISBN: 0-7073-0749-X.
3. Mezirow J. An overview on transformative learning. In: Illeris K, editor. Contemporary theories of learning: learning theorists in their own words: Routledge; 2009. p. 90-105.
4. Illeris K. A comprehensive understanding of human learning In: Illeris K, editor. Contemporary theories of learning: learning theorists in their own words: Routledge; 2009. p. 7-20.
5. Kolb DA. Experiential learning: Experience as the source of learning and development: FT press; 2014. ISBN: 0133892506.
6. Bandura A. Self-efficacy: toward a unifying theory of behavioral change. Psychol Rev. 1977;84(2):191.
7. Vygotsky LS. Mind in society: The development of higher psychological processes: Harvard university press; 1980. ISBN: 0674076680.
8. Dewey J. How we think: Courier Corporation; 1997. ISBN: 0486298957.
9. Lieberman DA, Bates CH, So J. Young children's learning with digital media. Computers in the Schools. 2009;26(4):271-83.
10. Mitchell A, Savill-Smith C. The use of computer and video games for learning; A review of the literature. London: Learning and Skills Development Agency; 2004. 93 p. ISBN: 1-85338-904-8.
11. Roschelle JM, Pea RD, Hoadley CM, Gordin DN, Means B. Changing How and What Children Learn in School with Computer-based Technologies. Future Child. 2000 09/01/;10(2):76-101. PMID: EJ625833.
12. Yelland N. The Future Is Now: A Review of the Literature on the Use of Computers in Early Childhood Education (1994-2004). AACE Journal. 2005 07/01/;13(3):201-32. PMID: EJ846362.
13. Roussou M. Learning by doing and learning through play: an exploration of interactivity in virtual environments for children. Computers in Entertainment (CIE). 2004;2(1):10-.

14. Fernandes S, Arriaga P, Esteves F. Using an Educational Multimedia Application to Prepare Children for Outpatient Surgeries. Health Commun. 2015;30(12):1190-200. PMID: 25144403. doi: 10.1080/10410236.2014.896446.
15. Fortier MA, Bunzli E, Walthall J, Olshansky E, Saadat H, Santistevan R, et al. Web-based tailored intervention for preparation of parents and children for outpatient surgery (WebTIPS): formative evaluation and randomized controlled trial. Anesth Analg. 2015 Apr;120(4):915-22. PMID: 25790213. doi: 10.1213/ANE.0000000000000632.
16. Jaaniste T, Hayes B, von Baeyer CL. Providing children with information about forthcoming medical procedures: A review and synthesis. Clinical Psychology: Science and Practice. 2007;14(2):124-43. PMID: 2007-08190-008. doi: 10.1111/j.1468-2850.2007.00072.x.
17. Kain ZN, Fortier MA, Chorney JM, Mayes L. Web-based tailored intervention for preparation of parents and children for outpatient surgery (WebTIPS): development. Anesth Analg. 2015 Apr;120(4):905-14. PMID: 25790212. doi: 10.1213/ANE.0000000000000610.
18. Lewis D. Computer-based approaches to patient education: a review of the literature. J Am Med Inform Assoc. 1999 Jul-Aug;6(4):272-82. PMID: 10428001.
19. Selander S. Designs for learning and ludic engagement. Digital Creativity. 2008;19(3):145-52.
20. Piaget J, Cook M. The origins of intelligence in children: International Universities Press New York; 1952.
21. Marton F, Trigwell K. Variatio est mater studiorum. Higher Education Research & Development. 2000;19(3):381-95.
22. Chauvet S, Hofmeyer A. Humor as a facilitative style in problem-based learning environments for nursing students. Nurse Educ Today. 2007 May;27(4):286-92. PMID: 16839647. doi: 10.1016/j.nedt.2006.05.008.
23. Hounsell D, McCune V, Hounsell J, Litjens J. The quality of guidance and feedback to students. Higher Education Research & Development. 2008;27(1):55-67.
24. Boud D, Molloy E. Feedback in higher and professional education: understanding it and doing it well: Routledge; 2013. ISBN: 0415692288.
25. Council NR. How people learn: Brain, mind, experience, and school: Expanded edition: National Academies Press; 2000. ISBN: 0309070368.
26. Buytendijk FJJ. Wesen und Sinn des Spiels : das Spielen des Menschen und der Tiere als Erscheinungsform der Lebenstriebe. Berlin: K. Wolff Verlag ; Der Neue Geist Verlag; 1933.
27. Boud D, Molloy E. Decision-making for feedback. In: Boud D, Molloy E, editors. Feedback in higher and professional education: understanding it and doing it well: Routledge; 2013.
28. Kamil ML, Intrator SM, Kim HS. The effects of other technologies on literacy and literacy learning. Handbook of reading research. 2000;3:771-88.
29. Andrews G, Woodruff E, MacKinnon KA, Yoon S. Concept development for kindergarten children through a health simulation. J Comput Assisted Learn. 2003;19(2):209-19. PMID: 2003-06095-007. doi: 10.1046/j.0266-4909.2003.00021.x.

30. Clements DH, Sarama J. Strip Mining for Gold: Research and Policy in Educational Technology-A Response to "Fool's Gold.". Educational Technology Review. 2003 01/01/;11(1). PMID: EJ673505.

31. Clements DH, Sarama J. Young Children and Technology: What's Appropriate? Yearbook (National Council of Teachers of Mathematics). 2005 01//;67:51.
